# Postoperative Hearing Outcomes and Usefulness of Endoscopy-Assisted Tympanoplasty in Pars Tensa Cholesteatoma

**DOI:** 10.1055/s-0044-1792016

**Published:** 2025-01-10

**Authors:** Takaomi Kurioka, Kunio Mizutari

**Affiliations:** 1 Department of Otorhinolaryngology. Head and Neck Surgery, National Defense Medical College, Saitama, Japan

**Keywords:** middle ear, cholesteatoma, tympanoplasty, hearing loss, endoscopy

## Abstract

**Introduction** In recent years, transcanal endoscopic ear surgery (TEES) has gained widespread recognition as an excellent surgical field for blind spots such as the sinus tympani (ST) when compared to microscopic ear surgery (MES).

**Objective** To investigate the postoperative hearing results for pars tensa cholesteatoma and the indications for utilizing endoscopy.

**Methods** The medical records of 16 patients (10 men and 6 women) with pars tensa cholesteatoma, who received initial surgical treatment between 2018 and 2022, were reviewed. We performed MES, TEES, or endoscopy-assisted MES (dual approach) depending on the pathological involvement in the mastoid cavity and ST.

**Results** The mean age of the patients was 45 years, and the surgical techniques utilized were MES in 2 cases, TEES in 7 cases, and dual approach in 7 cases. The preoperative pathological classification was stage I in 3 patients and stage II in 13 patients. The overall surgical success rates of postoperative hearing outcomes were 69% and 50% (1/2 patients) in the TEES group, 71% (5/7 patients) in the MES group, and 71% (5/7 patients) in the dual approach group. The successful cases (n = 11) were significantly younger and demonstrated better mastoid pneumatization than unsuccessful cases (n = 5).

**Conclusion** Endoscopy-assisted MES is appropriate for treating pars tensa cholesteatoma when pathological involvement is present at the deep bottom of the ST. Early surgical intervention and good eustachian tube function are crucial for improving hearing prognosis. Transcanal endoscopic ear surgery can be particularly useful in identifying and removing residual cholesteatoma within the ST.

## Introduction and Objective

Pars tensa cholesteatoma arises from retraction of the posterior-superior quadrant of the tympanic membrane (TM) and can easily involve the ossicular chain, resulting in significant hearing loss. 1 Furthermore, postoperative recurrence of TM retraction is commonly observed, resulting in poor hearing outcomes. 2 Despite the development of various surgical techniques, poor postoperative hearing outcomes have been reported in cases of pars tensa cholesteatoma. 3

Recently, the utility of transcanal endoscopic ear surgery (TEES) has been widely explored. 4 5 Compared with conventional microscopic ear surgery (MES), TEES offers a superior field of view and it is a minimally-invasive surgery that has been adopted by many institutions as a safe and established otologic procedure. 6 To date, the field of view of the microscope has been partially restricted, indicating several anatomical blind spots, such as the sinus tympani (ST). 7 As viewing the bottom of the ST directly during MES is particularly challenging, treating pars tensa cholesteatomas can be difficult. 8 The ST is the medial space to the pyramidal eminence, stapedius muscle, facial nerve (FN), and lateral to the posterior semicircular canal. Although access to this area during middle ear surgery in cases of pars tensa cholesteatoma is notoriously difficult, endoscopy is potentially helpful for the observation of and intervention in ST lesions. A recent study reported that pars tensa cholesteatoma is an aggressive disease, which presented high occurrence of residual disease, especially in the ST region, following MES. 9 10 This finding suggests that the TEES approach is advantageous for identifying residual disease and manipulating pathological lesions in pars tensa cholesteatoma. Despite the development of TEES, reports on its application to parse tensa cholesteatoma and its postoperative hearing outcomes are few. In the present study, we retrospectively evaluated the postoperative results of pars tensa cholesteatomas treated with TEES and discussed the indications for endoscopic surgery based on disease progression.

## Methods

### Patients

We retrospectively reviewed the medical records of patients with pars tensa cholesteatoma who received surgical treatment between 2018 and 2022 at National Defense Medical College Hospital. The study protocol was approved by the Institutional Review Board of National Defense Medical College (approval no.: 3103). Written informed consent was obtained from all participants.

### Surgical Treatment

The surgery was performed under general anesthesia. The MES included canal wall-up tympanoplasty with posterior tympanotomy when the lesion extended into the mastoid cavity. Single-stage surgery was primarily selected for intact canal wall tympanoplasty, although a staged operation was determined in cases with a high risk of residual disease. If the cholesteatoma is accompanied by severe infection, complete resection may not be accomplished because of the invisible epithelium's involvement. The ossicular chain was reconstructed using auricular cartilage, and cartilage tympanoplasty was performed to reinforce the TM using the auricular or tragus cartilage.

Transcanal endoscopic ear surgery is indicated when the lesion has not extended into the mastoid cavity, and when no pathological involvement at the bottom of the ST is observed. In cases involving the mastoid cavity and the bottom of the ST, we performed a dual approach of endoscopy-assisted MES. In the TEES procedure, 0- or 45-degree angled rigid endoscopes with an outer diameter of 2.7 mm (Karl Storz SE & Co. KG, Tuttlingen, Germany) and a full high-definition video system (Karl Storz SE & Co. KG) were used. Transcanal atticoantrotomy was performed to expose the entire cholesteatoma structure, and the cholesteatoma matrix was gently elevated from the surrounding tissues. If the cholesteatoma involved the incus or stapes, it was removed, and ossiculoplasty and cartilage tympanoplasty were performed using either cartilage or a hydroxyapatite prosthesis.

### Pathological Classification

The patients were classified according to the European Academy of Otology and Neurotology/Japanese Otological Society (EAONO/JOS) staging system. The cholesteatoma extension in each ear was surgically confirmed and scored according to middle ear involvement using the STAM system. Mastoid pneumatization was assessed using preoperative computed tomography (CT) and classified into 1 of 4 degrees according to the 2015 JOS staging system. The pathological status of the stapes was intraoperatively evaluated and classified into 2 groups according to the 2015 JOS staging system.

### Computed Tomography Examination and Classification

**Fig. 1:**
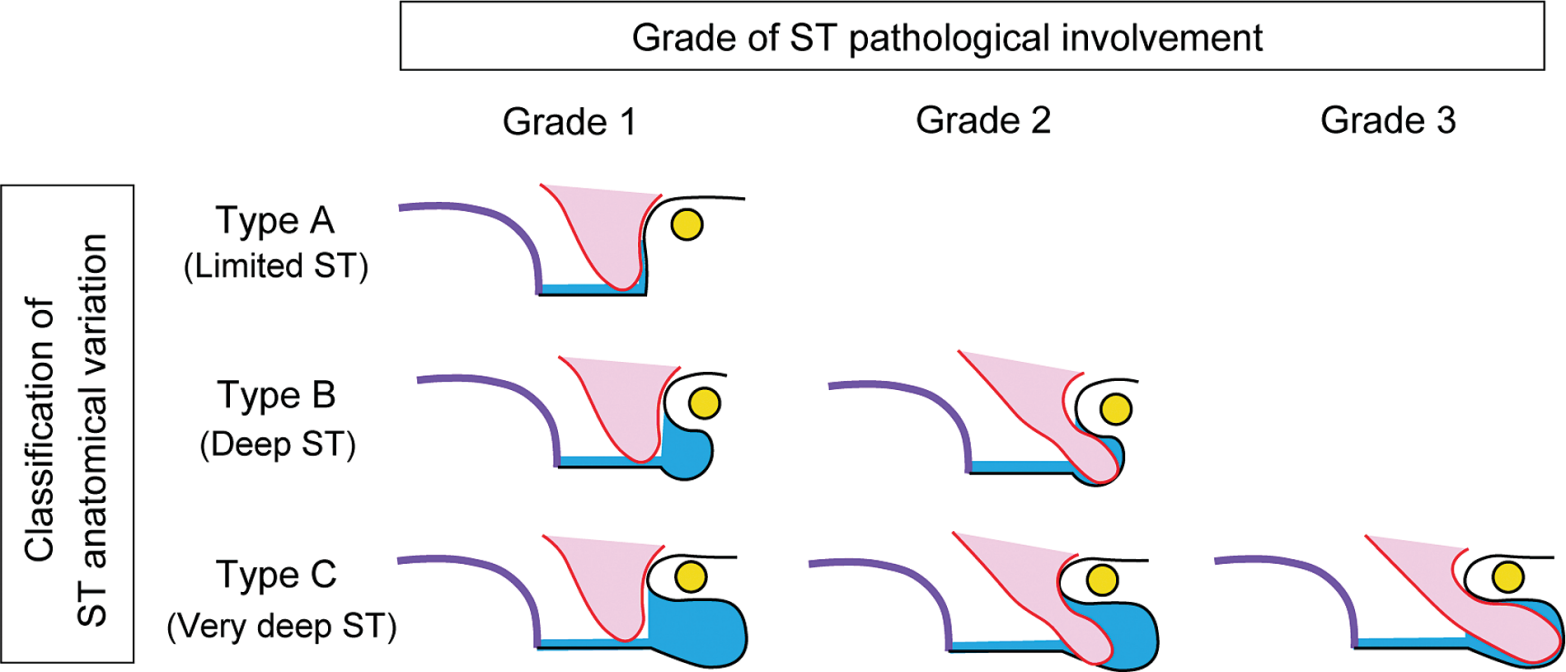
Grade of sinus tympani (ST) pathological involvement. In anatomical variations of the ST (blue area), type A (limited) is characterized by ST not extending to the mastoid segment of the facial nerve (FN, yellow circle). In type B (deep), the ST extends to the mastoid segment of the FN but without posterior extension, and in type C (very deep), the ST extends to the posterior limit of the FN. Regarding the grade of ST pathological involvement, grade 1 is defined as the cholesteatoma lesion (red area) that does not reach the mastoid segment of the FN. In grade 2, the lesion reaches the mastoid segment of the FN but does not go beyond there. In grade 3, the lesion extends to the mastoid segment of the FN. Purple line, promontory, ST, sinus tympani.

The anatomical classification of the ST was previously reported and is now divided into three categories. 11 12 Type A is defined as cases in which the bottom of the ST does not extend as deep as the FN; type B is defined as cases in which the bottom of the ST extends as far as the FN, and type C is defined as cases in which the bottom of the ST extends deeper than the FN. To evaluate the pathological involvement of the ST, we modified this classification ( Fig. 1 ). Grade 1 was defined as the bottom of the ST lesion that did not reach the mastoid segment of the FN. In grade 2, the bottom of the ST lesion reaches the mastoid segment of the FN but does not extend beyond it. In grade 3, the bottom of the ST lesion extended to the mastoid segment of the FN. In grade 1, TEES is applicable in almost all cases without mastoid invasion. However, for grades 2 and 3, a dual approach is often performed in our facility.

### Hearing Function

The hearing tests were conducted in a soundproof environment using an audiometer (AA-M1B; Rion, Tokyo, Japan). Hearing thresholds were acquired through air and bone conduction measurements at 0.25, 0.5, 1, 2, and 4 kHz. Sound masking was employed for the untested ear to prevent cross-hearing, whereas the other ear was tested as required. Audiogram data were used to determine postoperative hearing outcomes 1 year after middle ear surgery. Postoperative hearing results were classified as follows: air-bone gap (ABG) ≤ 10 dB, 20 dB, and 30 dB. We further divided the cases into two groups; “successful” was defined as postoperative ABG ≤ 20 dB, and “unsuccessful” was defined as postoperative ABG > 20 dB. To investigate the potential factors influencing on the postoperative hearing function in patients with pars tensa cholesteatoma, we analyzed the clinical and surgical features, including age, pathological staging, surgical approach, mastoid pneumatization, mastoid involvement, stapes invasion, ST pathological involvement, type of reconstruction, and cartilage tympanoplasty, between successful and unsuccessful cases.

## Statistical Analyses

Statistical analyses were performed using the JMP 14.2 software (SAS Institute Japan Inc., Tokyo, Japan). Chi-squared and nonparametric Mann-Whitney U tests were performed to examine clinical characteristics. Statistical significance was set at 0.05.

## Results

### Clinical Characteristics and Surgical Approaches

In total, there were 16 cases (10 men and 6 women) with pars tensa cholesteatoma with the first surgical treatment between 2018 and 2022. The clinical characteristics of the enrolled patients are presented in Table 1 . The mean patient age was 45 years. The surgical techniques utilized were MES in two cases, TEES in seven cases, and dual approaches in seven cases. The preoperative pathological classification was stage I in three patients and stage II in the remaining 13 patients. Next, pathological characteristics in each surgical approach were investigated ( Table 2 ). Age, pathological stage, and stapes invasion of cholesteatoma were not significantly different among the surgical approaches. As expected, pathological involvement in the mastoid cavity and ST were significantly higher in the dual approach group than in the TEES and MES groups ( *p < 0.05* ), which is consistent with the surgical indication in our facility. Staged surgery was performed in four cases.

**Table 1:** Clinical characteristics

Parameters	n
Age (mean ± SD; years)	45.0 ± 6.1
Sex (male/female; n)	10/6
Laterality (left/right; ears)	8/8
Staging (I/II; ears)	3/13
Surgical approach (TEES/MES/dual; ears)	7/2/7

**Abbreviations:** MES, microscopic ear surgery; SD, standard deviation; TEES, transcanal endoscopic ear surgery.

**Table 2:** Clinical characteristics in different surgical approaches

Parameters	TEES	MES	Dual	*p*
Age (mean, years)	46	68	38	0.34
Staging (I/II)	1/6	1/1	2/5	0.56
Mastoid pneumatization (without / with)	7/0	1/1	3/4	0.02*
ST pathological involvement (grade 1 / 2, 3)	5/2	1/1	0/7	0.02*
Stapes invasion (S0, 1 / S2, 3)	4/3	1/1	5/2	0.79

**Abbreviations:** MES, microscopic ear surgery; ST, sinus tympani; TEES, transcanal endoscopic ear surgery.

Note:*p < 0.05.

### Factors Affecting Postoperative Hearing Outcomes

We investigated the clinical factors affecting postoperative ABG ( Table 3 ). When examining whether the postoperative ABG was within 10 dB, the hearing outcomes were significantly better in patients 50 years of age and younger, in those without involvement in the mastoid cavity, and in those who have undergone cartilage tympanoplasty ( *p < 0.05* ). When the ABG ≤ 20 dB was examined, the results were significantly better in patients who were 50 years of age and younger and had good mastoid pneumatization. Finally, the ABG ≤ 30 dB was examined, and the results were significantly better in patients who were 50 years of age and younger. By contrast, pathological stage, surgical approach, stapes involvement, ST involvement, and the methods of ossicular chain reconstruction had no significant impact on postoperative ABG.

**Table 3:** Factors affecting postoperative air–bone gap

	Number of ears	Air–bone gap (dB)		
		≤10	≤20	≤30
Age				
≤ 50 years old	9	44%	100%	100%
> 50 years old	7	29%	29%	57%
Staging				
Stage I	4	50%	75%	75%
Stage II	12	33%	67%	83%
Surgical approach				
TEES	7	43%	71%	71%
MES	2	50%	50%	50%
Dual	7	29%	71%	100%
Mastoid pneumatization				
Good	5	40%	100%	100%
Poor	11	36%	55%	73%
Mastoid involvement				
Without	5	55%	82%	82%
With	11	0%	40%	80%
Stapes invasion				
S0, 1	11	36%	64%	73%
S2, 3	5	40%	80%	100%
ST pathological involvement				
Grade 1	8	50%	88%	88%
Grade 2, 3	8	25%	50%	75%
Reconstruction				
Type I	2	50%	50%	50%
Type III	7	43%	71%	86%
Type IV	7	29%	71%	86%
Cartilage tympanoplasty				
Without	11	18%	55%	64%
With	5	80%	100%	100%

**Abbreviations:** MES, microscopic ear surgery; ST, sinus tympani; TEES, transcanal endoscopic ear surgery.

Notes:*p < 0.05; **p < 0.01 *.*

Next, to evaluate the operative success rates, cases were classified into two groups. “Successful” group was characterized by a postoperative ABG of ≤ 20 dB, and “unsuccessful” group had a postoperative ABG of > 20 dB ( Table 4 ). The overall success rates of postoperative hearing outcomes were 69% and 71% (5/7 patients) in the TEES group, 50% (1/2 patients) in the MES group, and 71% (5/7 patients) in the dual-approach group. Significant differences were observed between the successful and unsuccessful groups in terms of mean age and mastoid pneumatization, with younger age and better mastoid pneumatization resulting in better hearing outcomes ( *p < 0.05* ). In addition, TEES and dual approaches did not demonstrate any superiority, regarding postoperative hearing outcomes, to the MES approach, indicating that the surgical technique employed did not significantly impact postoperative hearing outcomes. Other findings included a trend toward better hearing outcomes in cases of stage I ( *p = 0.11* ), without mastoid involvement ( *p = 0.09* ), with shallow ST involvement ( *p = 0.10* ), and with cartilage tympanoplasty ( *p = 0.07* ).

**Table 4:** Factors affecting surgical outcomes

Parameters	Successful (n = 11; 69%) (A–B gap ≤ 20 dB)	Unsuccessful (n = 5; 31%) (A–B gap > 20 dB)	*p*
Age (mean, years)	34.5	68.2	0.005**
Staging (I/II)	3/8	1/4	0.11
Surgical approach (TEES/MES/dual)	5/1/5	2/1/2	0.84
Mastoid pneumatization (good/poor)	5/6	0/5	0.03*
Mastoid involvement (without/with)	2/9	3/2	0.09
Stapes invasion (S0, 1/S2, 3)	7/4	4/1	0.51
ST pathological involvement (grade 1/2, 3)	7/4	1/4	0.10
Reconstruction (type I type III/type IV)	1/5/5	1/2/2	0.84
Cartilage tympanoplasty (without/with)	6/5	5/0	0.07

**Abbreviations:** A–B, air–bone; MES, microscopic ear surgery; ST, sinus tympani; TEES, transcanal endoscopic ear surgery.

Note:*p < 0.05 *.*

## Discussion

In the current study, we investigated the postoperative hearing outcomes of patients with pars tensa cholesteatoma according to surgical approach and pathological involvement. Hence, mastoid involvement, mastoid pneumatization, and young age are potentially valuable indicators of postoperative hearing outcomes. In addition, cartilage tympanoplasty would also be considered helpful for improving the postoperative results. However, in this study, the endoscopy-assisted dual approaches were comparable to the conventional MES approach in terms of postoperative hearing results, and no superiority in the usage of endoscopy was observed.

Since pars tensa cholesteatoma is often associated with adhesions in the posterior-superior quadrant of the TM, the middle ear mucosa at the pathological site is difficult to preserve during cholesteatoma dissection. 13 In particular, maintaining an air-containing space around the ossicles postoperatively is difficult, thus resulting in poor postoperative hearing improvement. 14 In addition, patients with pathological involvement in the ST or other blind spots are more likely to have residual lesions, indicating that a better surgical approach is required. 8 In recent years, TEES has been widely applied to different types of middle ear diseases. Transcanal endoscopic ear surgery has many advantages, including a wide field of view, higher magnification of fine anatomical structures, and clear visualization of anatomical areas in blind spots when viewed through a microscope. Therefore, we focused on endoscopy-assisted MES, which would be particularly useful in identifying and removing residual cholesteatoma located within the ST.

Regarding the location of cholesteatoma lesions, most pars flaccid cholesteatomas extend from the upper tympanic cavity to the mastoid cavity in the protympanum, tympanum, attic, and mastoid (PTAM) classification. All cases of pars tensa cholesteatoma contain T classification and then easily extend to the attic, mastoid, and anterior tympanic cavities. Thus, pars tensa cholesteatoma tends to extend to multiple subregions, suggesting that eustachian tube dysfunction is more strongly involved in addition to the narrowing and obstruction of the ventilation pathway in the middle ear cavity. 15 The higher prevalence of covert disease in pars tensa cholesteatomas is related to the formation pathway. 9 This unique pars tensa cholesteatoma development mode should be considered when planning middle ear surgery.

In the present study, hearing improvement outcomes were significantly worse in patients with poor mastoid pneumatization. This may be because patients with good eustachian tube function often have better mastoid pneumatization, which is less likely to recur postoperatively. Similar to the present results, the classification of mastoid pneumatization has been reported to be a useful predictive indicator of postoperative hearing outcomes. 13 However, some studies have reported that the degree of disease progression of pars tensa cholesteatoma does not correlate with postoperative hearing outcomes in contrast to pars flaccid cholesteatoma, indicating that pars flaccid cholesteatoma and pars tensa cholesteatoma should be considered separately. 16 Furthermore, in addition to the classification of mastoid pneumatization, our study demonstrated that age is a potentially helpful indicator of postoperative hearing outcomes. This supports the idea that shorter disease duration might be associated with better postoperative hearing outcomes, indicating that early treatment may be recommended for pars tensa cholesteatoma.

As for stapes lesions, 43% of the patients (6 ears) had S2 or higher, and the percentage of patients with stapes lesions was relatively high. Pars tensa cholesteatoma exhibited destruction of the stapes structure from an early stage, supporting a previous report that pars tensa cholesteatoma is more likely to cause hearing impairment than pars flaccid cholesteatoma. Type IV ossicular reconstruction is required for these cases with stapes involvement, and it has been previously reported that postoperative hearing performance tends to worsen. 17 However, in the present study, no relationship between hearing performance and presence or absence of stapes lesions or reconstruction method was identified. This may be because we actively used an endoscope in these stapes-involved cases to confirm that the columella was securely attached to the stapes footplate, and the results were better than those of previous reports. Therefore, otologic endoscopy is considered a useful tool that not only observes and removes pars tensa cholesteatoma lesions but also empowers us in the reconstruction of the ossicular chain.

Observation at the ST, which is often a blind spot during MES procedures, is possible in combination with endoscopy, which provides an excellent view and treatment option. In support of this concept, we occasionally experience cases in which microscopically seemingly complete removal has been achieved; however, when observed through an oblique endoscope, residual lesions are left at the bottom of the ST. The present study's overall success rate of postoperative hearing was 69%, which is consistent with previous reports. 15 The choice of the appropriate surgical procedure based on the classification of pathological progression, cartilage tympanoplasty, and endoscopic ossiculoplasty may be effective. However, the postoperative hearing results were still not satisfactory, and further study and improvement of the surgical technique are needed.

Finally, our findings have important clinical implications for the treatment of pars tensa cholesteatomas. The indications for endoscopic surgery for pars tensa cholesteatoma at our institution are limited to those without mastoid involvement. However, recent advances in powered TEES using ultrasonic bone retractors and curved bars have expanded its indications for mastoid lesions. Furthermore, pars tensa cholesteatoma requires long-term observation, of at least 5 to 10 years after the last surgery, for residual disease to be detected as recurrence. 18 Since the purpose of the present study was to evaluate the postoperative hearing outcomes, not recurrence, we assessed the hearing threshold at 1 year postoperatively as a relatively short-term result. Future studies are warranted to investigate the long-term changes in postoperative hearing outcomes and recurrence rates. Finally, this was a single-hospital retrospective study with a relatively small sample size; therefore, additional studies with larger sample sizes are needed.

## Conclusion

Endoscopy-assisted MES is appropriate for treating pars tensa cholesteatoma when pathological involvement is present at the deep bottom of the ST. Early surgical intervention and good eustachian tube function are crucial for improving hearing prognosis. Transcanal endoscopic ear surgery can be particularly useful in identifying and removing residual cholesteatoma within the ST.
